# Herbal Medicines for Inflammatory Diseases

**DOI:** 10.1155/2014/982635

**Published:** 2014-12-24

**Authors:** Seong-Gyu Ko, Chang Shik Yin, Bing Du, KyoungHyun Kim

**Affiliations:** ^1^Laboratory of Clinical Biology and Pharmacogenomics and Center for Clinical Research and Genomics, Kyung Hee University, Seoul 130-701, Republic of Korea; ^2^Acupuncture and Meridian Science Research Center, College of Korean Medicine, Kyung Hee University, Seoul, Republic of Korea; ^3^Shanghai Key Laboratory of Regulatory Biology, School of Life Science, Institute of Biomedical Sciences, East China Normal University, Shanghai, China; ^4^Division of Environmental Genetics and Molecular Toxicology, Department of Environmental Health, College of Medicine, University of Cincinnati, Cincinnati, OH 45267, USA

Inflammation plays an essential role in the development of various human diseases including asthma, rheumatoid arthritis, inflammatory bowel disease, Crohn's disease, and tendonitis. Moreover, chronic inflammatory response is a major driving force for the progression of cancer, atherosclerosis, diabetes, obesity, and Alzheimer's disease. When under control, inflammatory process is beneficial in protecting the body from disastrous breakdown whereas inflammatory process is harmful, when unrestrained, resulting in unwanted breakdown of the body. In this sense, inflammation can be a two-edged sword.

Herbal medicines have long been used for preventing or treating diseases including inflammatory diseases and indeed a priceless source of valuable chemical compounds that developed into indispensable drugs in medical practice. Nonetheless, their valuable effects in inflammatory diseases have not been thoroughly investigated. Today, there are plenty of chemical drugs in the shelves of the pharmacy. We may take those modern chemical drugs for granted. However, herbal medicine has been with us from prehistoric days as a rich source of medicinal compounds. Herbal medicine is a valuable constituent of traditional medicine and modern medicine, as will be probably the same in the future.

Considering that inflammation is one of the essential factors in the development of many human diseases, there is an urgent need to expand our scientific understanding for beneficial effects of herbal medicines on inflammatory diseases. Yes, it is true that there is a long way to go to elucidate precise effects of herbal medicines at molecular level. However, it is time to be more aware of the importance and potential of herbal medicine in inflammatory process, considering rapidly expanding its use and application.

This special issue will introduce you to the valuable research reports on herbal medicine in inflammatory diseases, ranging from basic researches to explore roles of herbal medicines against inflammatory diseases, to clinical trials to access roles of herbal medicines for inflammatory diseases, to combinatorial uses of herbal medicines with conventional treatment for inflammatory diseases, to analyses of bioactive compounds in medicinal plant extracts for inflammatory diseases, and to systematic reviews for inflammatory diseases. We hope this timely special issue be a valuable resource to contemporary outstanding researchers.

*Seong-Gyu Ko* *Seong-Gyu Ko* *Chang Shik Yin* *Chang Shik Yin* *Bing Du* *Bing Du* *KyoungHyun Kim* *KyoungHyun Kim*

